# Metagenomic analysis through the extended Burrows-Wheeler transform

**DOI:** 10.1186/s12859-020-03628-w

**Published:** 2020-09-16

**Authors:** Veronica Guerrini, Felipe A. Louza, Giovanna Rosone

**Affiliations:** 1 Dipartimento di Informatica, Università di Pisa, Largo B. Pontecorvo, 3, Pisa, Italy; 2 Faculty of Electrical Engineering, Federal University of Uberlândia, Uberlândia, Brazil

**Keywords:** Metagenomics, Next-generation sequencing, Classification, Alignment-free, Assembly-free, eBWT, LCP Array

## Abstract

**Background:**

The development of Next Generation Sequencing (NGS) has had a major impact on the study of genetic sequences. Among problems that researchers in the field have to face, one of the most challenging is the taxonomic classification of metagenomic reads, i.e., identifying the microorganisms that are present in a sample collected directly from the environment. The analysis of environmental samples (metagenomes) are particularly important to figure out the microbial composition of different ecosystems and it is used in a wide variety of fields: for instance, metagenomic studies in agriculture can help understanding the interactions between plants and microbes, or in ecology, they can provide valuable insights into the functions of environmental communities.

**Results:**

In this paper, we describe a new lightweight alignment-free and assembly-free framework for metagenomic classification that compares each unknown sequence in the sample to a collection of known genomes. We take advantage of the combinatorial properties of an extension of the Burrows-Wheeler transform, and we sequentially scan the required data structures, so that we can analyze unknown sequences of large collections using little internal memory. The tool LiME (Lightweight Metagenomics via eBWT) is available at https://github.com/veronicaguerrini/LiME.

**Conclusions:**

In order to assess the reliability of our approach, we run several experiments on NGS data from two simulated metagenomes among those provided in benchmarking analysis and on a real metagenome from the Human Microbiome Project. The experiment results on the simulated data show that LiME is competitive with the widely used taxonomic classifiers. It achieves high levels of precision and specificity – e.g. 99.9*%* of the positive control reads are correctly assigned and the percentage of classified reads of the negative control is less than 0.01*%* – while keeping a high sensitivity. On the real metagenome, we show that LiME is able to deliver classification results comparable to that of MagicBlast. Overall, the experiments confirm the effectiveness of our method and its high accuracy even in negative control samples.

## Background

Interest in metagenomics has been sparked by the recent advances in “next-generation” DNA sequencing (NGS) technologies [1]. Metagenomics refers to the sequencing of microbial DNA collected directly from the environment, without isolation and lab cultivation of individual species. The analysis of environmental samples (i.e., metagenomes) are particularly important to figure out the microbial composition of different ecosystems and it is used in several fields: for example, metagenomic studies in agriculture are being used to explore the relations between microbes and plants and to detect crop diseases. Of fundamental importance is to identify with precision the microorganisms that are present in a metagenomic sample by comparing the biological sequences therein and assigning them to a specific taxon.

The metagenomic tools can be preliminarily classified into two categories: reference-based (supervised) [2–7] and reference-free (unsupervised) [8, 9]. In the first case, the metagenomic analysis needs reference genomes and the goal is to match sequences, typically reads or assembled contigs, against a database of microbial genomes in order to identify the taxon of each sequence. In the second case, the aim is to subdivide the reads or the sequences assembled from metagenomic reads into discrete units, without the need of references, so that sequences clustered together display individual populations that comprise the microbial community. This latter approach is known as reference-free binning [8].

In this paper, we focus on the first approach which provides taxonomic classification of individual sequences from a metagenomic sample. A variety of strategies have been used for the matching step of the reference-based metagenomics tools. The following partial list gives a few examples: aligning reads, mapping k-mers, using complete genomes, aligning marker genes only or translating the DNA and aligning to protein sequences (see also [10]). One can split these methods into alignment-based and assembly-based approaches or alignment-free and assembly-free approaches [11].

Alignment-based classifiers proceed by aligning metagenome sequences against the genomes in a reference database. The most well-known and commonly used tool for DNA search and alignment is BLAST (Basic Local Alignment Search Tool) [12]. BLAST consists of a set of algorithms that attempt to find a short fragment of a query read that aligns with a fragment of a genome stored in the reference database.

Nevertheless, as reference databases and NGS sequencing datasets have grown in size, this alignment strategy has become computationally expensive and alignment-free methods have been developed. So, several techniques exist to reduce the computational complexity of this approach. In particular, there exist many statistical/computational tools for metagenomic classification (e.g. [2–4, 13]), many of these approaches use exact matching of short words of length *k* (k-mers) rather than alignment and are often based on hashing, indexing and counting k-mers.

Recent surveys (for instance [14, 15]) offer a thorough benchmarking analysis by comparing the majority of the state-of-the-art tools. A recent evaluation of tools for metagenome classification [14] presents the most widely used tools tested on complex and realistic datasets. According to this benchmarking analysis, Kraken [2] and CLARK [3] result to be top-performing tools in terms of both similarity to the correct answer and the fraction of reads classified. Note that these two tools are both *k*-mer based. An alternative approach to fixed *k*-mers is to use spaced seeds, i.e. patterns in which some fixed positions are allowed to be wild-cards. CLARK-S [5] is the new version of CLARK that uses spaced seeds^1^ and achieves higher sensitivity with the same high precision. The main drawback of these approaches is that they are extremely memory consuming: the memory usage of CLARK-S, as well as of CLARK and Kraken, is high, and the results obtained by running the lightweight version CLARK-L are indicated to be a “draft, or first order approximation” of those obtained by running CLARK or CLARK-S. Recently, a new version of Kraken, named Kraken 2 [7], has been introduced. It improves upon Kraken by reducing memory usage by 85%, and allowing to use greater amounts of reference genomic data while maintaining high accuracy. There are, however, other efficient alignment-free methods not based on *k*-mer counting, for instance Centrifuge [4], which is based on a read-mapping strategy and uses the FM-index [16] to store and index the genome database. Another recent tool based on mapping is TaxMaps [6]: it uses a database compression algorithm that eliminates redundancy by performing a lowest common ancestor (LCA) pre-assignment and collapse for k-mers of length greater than a specified read length; this allows GEM mapper [17] to conduct non-exact searches in the same manner as it would against the original database, resulting in compression that, for the purpose of taxonomic classification, is lossless. Finally, a novel member of the BLAST family of programs has been introduced recently: Magic-BLAST [18] is a new tool for mapping DNA or RNA next-generation sequencing (NGS) runs against a whole genome.

**Our contributions —** We propose a new alignment-free, mapping-free and assembly-free method for comparing sequences (cf. [11, 19, 20]), which is combinatorial by nature and allows us to use little internal memory with respect to other approaches, since the use of the internal memory mainly depends on the number of reads that one wants to examine at the same time.

Our method is based on an extension of the Burrows-Wheeler Transform (shortly eBWT) to a collection of sequences [21]. The eBWT has been used in several application contexts as the circular pattern matching (cf. [22]) and the alignment-free methods for comparing sequences (cf. [21, 23–26]). Different distance measures have been defined and successfully applied to several biological datasets, as for instance mitochondrial DNA genomes [21, 23], expressed sequence tags [27] and proteins [24].

Usually, when the eBWT is applied to a collection $\mathcal {S}$ of sequences, its output string $\textsf {ebwt}(\mathcal {S})$ is enriched by another data structure, called the document array $\textsf {da}(\mathcal {S})$: a different color is assigned to each element of $\mathcal {S}$ and each symbol of $\textsf {ebwt}(\mathcal {S})$ is associated with a color in $\textsf {da}(\mathcal {S})$ [28]. In other words, the array $\textsf {da}(\mathcal {S})$ contains a sequence of colors that depends on how the suffixes of the sequences in $\mathcal {S}$ are mixed in the sorted list. In [21, 23], the authors define a class of dissimilarity measures that, by using the eBWT, formalize the intuitive idea that the greater is the number of substrings shared by two sequences $u, v \in \mathcal {S}$, more their colors are intermixed in $\textsf {da}(\mathcal {S})$, and the smaller is the “distance” between *u* and *v*.

In this paper, we present a tool for the metagenomic classification task, called LiME (Lightweight Metagenomics via eBWT), that takes as input the (extended) Burrows-Wheeler Transform enhanced with the document array (DA) and the longest common prefix (LCP) array [29]. These are fundamental data structures in string processing and can be built independently from our tool (e.g. [30–35]).

The contribution of this paper is twofold, theoretical as well as practical. The theoretical result is the introduction of a novel combinatorial approach, which is alignment-free, mapping-free and assembly-free, that can be the basis of new biological sequence analysis methods. Our approach takes advantage of the combinatorial property of the Burrows-Wheeler Transform (BWT) [36], already exploited by BWT-based compressors (see, for instance [37–39]): the output of BWT shows a local similarity (occurrences of a given letter that precede the same context tend to occur in clusters) [40–43].

From a practical viewpoint, unlike other approaches, LiME does not need to build ad-hoc and keep in internal memory the data structures relating to the database of the reference genomes, as it only needs to run sequential scans on the input data structures, that may consist of a simple text file or may be in compression form. For the best of our knowledge, LiME also is the first metagenomic classifier that runs a many-to-many pairwise comparison and that is able to produce a similarity matrix, by comparing all unknown sequences in the sample versus all known genomes in the collection at the same time in order to be able to assign the correct reference to each read. In the future, such matrix could also be used for other metagenomic applications, for instance reference-free (unsupervised) approaches [9] or all-vs-all comparison of metagenomes [44].

Moreover, the experiments show that our tool has a very high precision and a high specificity, in fact our tool is able to correctly assign a read to a genome, while being able to establish that random reads must not be assigned to any genome. Our tool can take paired-end read collections as input - we recall that a paired-end read is a pair consisting of two reads (or mates), such that the second read occurs in the genome at a known distance after the first one. LiME is able to process the mates individually while still recognizing the pairing information, when the input set is a paired-end read.

This work is an extended version of a paper appeared in [45], where two of the present authors introduced a new similarity measure, based on the clustering effect of the eBWT. The main idea consists in computing the similarity between a read and a genome by identifying and analyzing the consecutive symbols in the output of the eBWT and their related colors in the DA. The previous strategy did not take into account the symbols in the International Union of Pure and Applied Chemistry (IUPAC)^2^ code [46], indeed they were considered as placeholders. Moreover, in the preliminary version, several reads were set as ambiguous, and thus not classified, by leaving a more in-depth analysis of them for a further work.

The additional contributions over those in the conference paper [45] are: i) we modify the analysis of the clusters by using two different similarity measure definitions: in the first variant, we count how many matches of symbols there are between each read and each genome in any cluster taking into account also the IUPAC ambiguity code (an ambiguity code can match with themselves or with any letter that is associated with its code); whereas, in the second variant, only the presence of such symbols in clusters is considered and we just distinguish the belonging to different input sequences; ii) we modify the read classification by doing a more in-depth analysis of the ambiguous reads (note that the classification is divided into three phases and only the first phase is in common with [45]); iii) we are able not only to classify the reads at a specific taxonomic rank such as genomes, or any level between species and phylum, but also to classify reads at several taxonomic levels by taking advantage of the taxonomic lineage; iv) we implement a multi-threading version of our tool exploiting the fact that it allows a certain degree of parallelization, indeed as stated in [45], the analysis of clusters is independent of each other, so each thread can handle distinct parts of the input files by reading it through local buffers; we also extend the experiments showing the performance of our tool on a real dataset.

The validation is performed by using both simulated metagenomes among those provided in the benchmarking analysis [14] and a real metagenome from the Human Microbiome Project (HMP).

Concerning the simulated datasets, they reproduce size, complexity and characteristics of real metagenomic samples containing around 20 millions of sequences (for the positive control) in addition to a negative control set of about 5 millions of random shuffled reads which mimic sequences from unknown organisms that are likely to appear in metagenomic samples. The experiment results on the simulated data show that LiME is competitive with the state-of-the-art tools. It achieves high levels of precision and specificity– e.g., 99.9*%* of the positive control reads are correctly assigned and the percentage of classified reads of the negative control is less than 0.01*%* – keeping a high sensitivity. Thus, on simulated datasets LiME achieves a high F1 score, which is the harmonic mean between sensitivity and precision. Recall that a classifier obtaining a high F1 score is able to achieve both high precision and high sensitivity. Moreover, LiME is able to deliver classification accuracy comparable to that of Magic-BLAST, TaxMaps and CLARK-S, yet requiring less computational cost.

On the real metagenome, the accuracy of classification cannot be evaluated, since the “ground truth” for real metagenome is not available, and thus we choose to compare the classification of LiME to the other tools’ classification results. By comparing the experimental results, we observe that LiME classifies identically the same reads as Magic-BLAST more than the other tools do. Moreover, the number of reads that are classified by LiME but not by Magic-BLAST is greater than that computed by CLARK-S, Centrifuge, and Kraken 2, although LiME classifies more reads than CLARK-S, Centrifuge and Kraken 2.

Overall, the experiments confirm the effectiveness of our method and its high accuracy even in negative control samples.

## Method

In this section, we present a new eBWT-based strategy to tackle the problem of metagenomic classification that is assembly-, mapping- and alignment-free and uses a little amount of memory, in the sense that we keep the input data structures we use in external memory, and the similarity matrix between the reads and the genomes in internal memory. So, the space in internal memory depends only on the number of sequences that one wants to examine at the same time and it does not depend on the size (number of symbols) of the input.

### Preliminaries and materials

Let *S* be a string (or sequence) of length *n*, and *Σ* its alphabet set, with *σ*=|*Σ*|. We denote the *i*-th symbol of *S* by *S*[ *i*]. Let $\mathcal {S}=\{S_{1},S_{2},\ldots,S_{m}\}$ be a collection of *m* strings. We assume that each string $S_{i} \in \mathcal {S}$ of length *n*_*i*_ is followed by a special symbol *S*_*i*_[*n*_*i*_+1]=*$*_*i*_, which is lexicographically smaller than any other characters in $\mathcal {S}$, and does not appear in $\mathcal {S}$ elsewhere — for implementation purposes, we may simply use a unique end-marker *$* for all strings in $\mathcal {S}$. The alphabet *Σ* is the biological alphabet {*A*,*C*,*G*,*T*} enhanced with the degenerated base symbols (IUPAC code [46]) and the end-marker symbol.

A *substring* of any $S \in \mathcal {S}$ is denoted as *S*[ *i*,*j*]=*S*[ *i*]⋯*S*[ *j*], with *S*[ 1,*j*] being called a *prefix* and *S*[*i*,*n*+1] a *suffix* of *S*. A *range* is delimited by a square bracket if the corresponding endpoint is included.

**Fig. 1:**
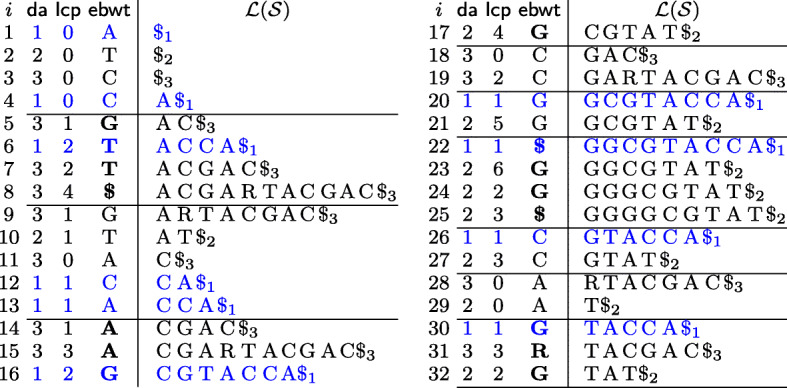
The required data structures for our running example. The set $\mathcal {S}$ is {*S*_1_=*GGCGTACCA$*_1_,*S*_2_=*GGGGCGTAT$*_2_,*S*_3_=*ACGARTACGAC$*_3_}, where *S*_1_ is a read and *S*_2_ and *S*_3_ are two genomes. The symbols of $\textsf {ebwt}(\mathcal {S})$ in the 2-clusters are in bold

The *Burrows-Wheeler Transform* (BWT) [36] is a well-known widely used reversible string transformation that can be extended to a collection of strings. Such an extension, known as eBWT or multi-string BWT, is a reversible transformation whose output string (denoted by $\textsf {ebwt}(\mathcal {S})$) is a permutation of the symbols of all strings in $\mathcal {S}$ [21] (see also [30, 31, 34, 47, 48]). The length of $\textsf {ebwt}(\mathcal {S})$ is denoted by $N=\left (\sum _{i=1}^{m}n_{i}\right) + m$, and $\textsf {ebwt}(\mathcal {S})[i]=x$, with 1 ≤ *i* ≤ *N*, if *x* circularly precedes the *i*-th suffix *S*_*j*_[*k*,*n*_*j*_+1] (for some 1≤*j*≤*m* and 1≤*k*≤*n*_*j*_ + 1), according to the lexicographic sorting of the suffixes of all strings in $\mathcal {S}$. In this case, we say that the suffix *S*_*j*_[*k*,*n*_*j*_+1] is associated with the position *i* in $\textsf {ebwt}(\mathcal {S})$ and with the color $j\in \{1, 2, \dots, m\}$ in the DA. Then, the output string $\textsf {ebwt}(\mathcal {S})$ is enhanced with the *document array* of $\mathcal {S}$ (denoted by $\textsf {da}(\mathcal {S})$) of length *N* where $\textsf {da}(\mathcal {S})[i]=j$, with 1≤*j*≤*m* and 1≤*i*≤*N*, if $\textsf {ebwt}(\mathcal {S})[\!i]$ is a symbol of the string $S_{j} \in \mathcal {S}$. In other words, a different color is assigned to each sequence. See Fig. 1 for an example.

The *longest common prefix* (LCP) array [29] of $\mathcal {S}$ is the array $\textsf {lcp}(\mathcal {S})$ of length *N*+1, such that $\textsf {lcp}(\mathcal {S})[\!i]$, with 2≤*i*≤*N*, is the length of the longest common prefix between the suffixes associated with the positions *i* and *i*−1 in $\textsf {ebwt}(\mathcal {S})$, and $\textsf {lcp}(\mathcal {S})[\!1] = \textsf {lcp}(\mathcal {S})[N+1]= 0$ by default.

The set $\mathcal {S}$ will be omitted if it is clear from the context. Moreover, for clarity of description, we denote by $\mathcal {L(S)}$ the sorted list of the suffixes in $\mathcal {S}$, distinguishing the end-marker symbol *$*, although we do not need it for our computation.

We call *u*-*occurrence* any substring *u* that occurs in any sequence of $\mathcal {S}$.

#### **Remark 1**

Recall that $\textsf {ebwt}(\mathcal {S})$ is implicitly associated with $\mathcal {L(S)}$ and all the suffixes in $\mathcal {S}$ starting with the same substring *u*, with |*u*|=*k*, must be consecutive entries in $\mathcal {L(S)}$ in the range [ *h*,*j*]. Moreover, *lcp*[ *h*]<*k*, *lcp*[*j*+1]<*k*, and lcp[ *i*]≥*k* for *i*=*h*+1,…,*j*, and the symbols of $\mathcal {S}$ that are followed by *u*-occurrences coincide with ebwt[ *h*,*j*].

#### **Remark 2**

Let *ℓ* be the total number of *u*-occurrences in $\mathcal {S}$, with |*u*|=*k*, there exist *k*−1 substrings (i.e., all suffixes of *u* that are not equal to *u*) which appear *at least* *ℓ* times in $\mathcal {S}$.

#### **Example 1**

(running example) Let $\mathcal {S}= \{S_{1}=GG\textbf {CGT}ACCA\$_{1}, S_{2}=GGGG\textbf {CGT}AT\$_{2}, S_{3}=ACGARTACGAC\$_{3}\}$. The substring *CGT* appears exactly once in sequences *S*_1_ and *S*_2_. The two suffixes of *S*_1_ and *S*_2_ starting with *CGT*-occurrences occupy consecutive positions, precisely *16* and *17* in Fig. 1, and lcp[ 17]=4. Moreover, according to Remark 2, we may note that the number of *GT*-occurrences is 2 and the one of *T*-occurrences is *4*.

### Preprocessing

We recall that our tool takes as input the files containing $\textsf {ebwt}(\mathcal {S})$, $\textsf {da}(\mathcal {S})$ and $\textsf {lcp}(\mathcal {S})$ of the collection $\mathcal {S}$ of reads and reference genomes.

Let $\mathcal {S}=\{S_{1},\ldots,S_{m}\}$ be the input collection of biological sequences comprising *r* reads and *g* genomes, where *m*=*r*+*g*. More in details, $S_{i} \in \mathcal {S}$ is a read if 1≤*i*≤*r* and $S_{j}\in \mathcal {S}$ is a genome if *r*+1≤*j*≤*m*. For simplicity, we denote by $\mathcal {R}$ the subset of reads and by $\mathcal {G}$ the subset of genomes.

The construction of $\textsf {ebwt}(\mathcal {S})$, $\textsf {da}(\mathcal {S})$ and $\textsf {lcp}(\mathcal {S})$ can be performed in internal or external memory. We can compute $\textsf {ebwt}(\mathcal {G})$ and $\textsf {da}(\mathcal {G})$ with algorithm GAP [30] or eGSA [47]. Then, for each new experiment with a read collection $\mathcal {R}$, we can compute $\textsf {ebwt}(\mathcal {R})$ and $\textsf {da}(\mathcal {R})$ by using the algorithm BCR [31, 32] and merge them with $\textsf {ebwt}(\mathcal {G})$ and $\textsf {da}(\mathcal {G})$ to compute $\textsf {ebwt}(\mathcal {S})$ and $\textsf {da}(\mathcal {S})$ by using the algorithm eGAP [34], which gives $\textsf {lcp}(\mathcal {S})$ as a by-product with no additional costs.

Notice that the used method does not affect the classification, so one can use other algorithms to construct $\textsf {ebwt}(\mathcal {S})$, $\textsf {da}(\mathcal {S})$ and $\textsf {lcp}(\mathcal {S})$ which is a good feature, since more efficient tools can appear in the literature.

### LiME

In this section, we describe how computing a similarity value between a short sequence and a genome reference, and then we describe how to handle the reverse complement strand of the sequence or the mates for paired-end read collections.

We outline the method that we introduced to classify short reads by assigning it to a specific taxon according to the similarity scores computed for any genome in $\mathcal {G}$ through sequential scans on $\textsf {ebwt}(\mathcal {S})$, $\textsf {da}(\mathcal {S})$ and $\textsf {lcp}(\mathcal {S})$.

Our notion of similarity between sequences exploits the underlying properties of the eBWT: (i) the clustering effect, i.e., the fact that this transformation tends to group together symbols that occur in similar contexts in the input string collection; (ii) the fact that if a substring *u* occurs in one or more sequences, then the suffixes of the collection starting with *u*-occurrence are likely to be close in the sorted list of suffixes. In other words, the greater the number of substrings shared by two sequences is, the more they are similar.

Roughly speaking, we consider the symbols of $\mathcal {S}$ followed by the same substrings (i.e., contexts) which are clustered together in $\textsf {ebwt}(\mathcal {S})$ and match one-to-one the symbols belonging to $\mathcal {R}$ to the symbols belonging to $\mathcal {G}$.

The overall scheme of the LiME algorithm can be sketched as follows: Step 1. By reading $\textsf {da}(\mathcal {S})$ and $\textsf {lcp}(\mathcal {S})$ in a sequential way, we detect *α*-clusters, i.e., the blocks of $\textsf {ebwt}(\mathcal {S})$ containing symbols belonging both to $\mathcal {R}$ and to $\mathcal {G}$ and whose associated suffixes in $\mathcal {L(S)}$ share a common context (*u*-occurrence) of a minimum length *α*; Step 2. We analyze *α*-clusters in order to evaluate a degree of similarity between any read and any genome in $\mathcal {S}$ by using two different approaches:

- by reading both $\textsf {da}(\mathcal {S})$ and $\textsf {ebwt}(\mathcal {S})$, and
- by reading only $\textsf {da}(\mathcal {S})$.

Step 3. We perform the read assignment: every read is assigned to a specific taxon either at a given taxonomic level (if it is specified) or at any level by taking advantage of the taxonomic lineage of taxa.

**Step 1: build*α*-clusters collection —** In Step 1, inspired by Remark 1, we build the collection $\mathcal {C}_{\alpha }$ of *α*-clusters, which are blocks of symbols delimited by a pair of indices.

#### **Definition 1**

Let *α* be a positive integer, lcp[1,*N*+1] be the LCP-array and da[1,*N*] the document array associated with ebwt[1,*N*]. An *α-cluster* of $\textsf {ebwt}(\mathcal {S})$ of size *pE*−*pS*+1 is any pair of indices (*pS*,*pE*) in [1,*N*] such that ∙ lcp[ *pS*]<*α*, and lcp[*pE*+1]<*α*, ∙ lcp[ *i*]≥*α*, for every *pS*<*i*≤*pE*, ∙ there exist two indices *s*,*t*, *pS*≤*s*,*t*≤*pE*, such that da[ *s*]≤*r* and da[ *t*]>*r*,

where *r* is the total number of reads in $\mathcal {S}$.

#### **Example 2**

(running example) By setting *r*=1 and *g*=2, we have $\mathcal {R}=\{S_{1}\}$ and $\mathcal {G}=\{S_{2},S_{3}\}$. For *α*=2, the set $\mathcal {C}_{2}$ of 2-clusters of the $\textsf {ebwt}(\mathcal {S})$ is given by {(5,8),(14,17),(20,21),(22,25),(26,27),(30,32)}, as depicted in Fig. 1.

In other words, we discard the blocks of $\textsf {ebwt}(\mathcal {S})$ whose associated suffixes do not share a prefix of length at least *α* or that contain symbols belonging only to one set ($\mathcal {R}$ or $\mathcal {G}$). Setting a minimum LCP *α* is to filter out blocks corresponding to short random contexts (*u*-occurrences) which are statistically not significant, while imposing symbols from both sets is appropriate for next step’s calculations. Moreover, since the genomes in $\mathcal {G}$ are (usually) long sequences, the parameter *α* must be chosen smaller than the read length (sequences in $\mathcal {R}$) to have $\mathcal {C}_{\alpha }$ not empty.

Step 1 can be performed by a sequential scan over $\textsf {lcp}(\mathcal {S})$ and $\textsf {da}(\mathcal {S})$ allowing us to keep the input data structures in external memory and use only a small amount of internal memory to detect *α*-clusters. Since the clusters do not overlap, we implemented LiME so that it performs this construction in parallel way, by dividing the data structures appropriately.

It is easy to see that we compute the similarity between a read $S_{i}\in \mathcal {R}$ and a genome $S_{j}\in \mathcal {G}$ by analyzing the data structures of the entire set of sequences $\mathcal {S}$, not only the two sequences *S*_*i*_ and *S*_*j*_. This is possible according to the following remark.

#### **Remark 3**

Let (*pS*,*pE*) be an *α*-cluster of $\textsf {ebwt}(\mathcal {S})$ that contains at least a symbol of *S*_*i*_ and at least a symbol of *S*_*j*_. Other symbols that belong to sequences in $\mathcal {S}$ apart from *S*_*i*_ and *S*_*j*_ may also appear in ebwt[*pS*,*pE*]. Nevertheless, we can implicitly get a new cluster (*pS*^′^,*pE*^′^) by deleting from the $\textsf {ebwt}(\mathcal {S})$ all symbols not belonging to *S*_*i*_ and *S*_*j*_, and for the properties of the LCP array, it is easy to verify that (*pS*^′^,*pE*^′^) forms an *α*-cluster of ebwt({*S*_*i*_,*S*_*j*_}).

**Step 2: build the similarity matrix —**We compute the $|\mathcal {R}| \times |\mathcal {G}|$ similarity matrix by analyzing the *α*-clusters detected in the previous step. The idea in this step is to use the information from the clustering effect in $\textsf {ebwt}(\mathcal {S})$ and the mixed colors in $\textsf {da}(\mathcal {S})$. Also in this step, we perform sequential scans of the input data structures.

During the second step, we analyze each *α*-cluster of the $\textsf {ebwt}(\mathcal {S})$ by using one of the two approaches:

- by reading both $\textsf {da}(\mathcal {S})$ and $\textsf {ebwt}(\mathcal {S})$,
- by reading only $\textsf {da}(\mathcal {S})$,

Then, we measure the degree of similarity between each sequence in $\mathcal {R}$ and each genome in $\mathcal {G}$.

In the first approach, whose related measure we call *α*^eBWT^-similarity, we consider the symbols that appear in the *α*-clusters by making an exact correspondence between the symbols belonging to $\mathcal {R}$ and $\mathcal {G}$ and taking into account the ambiguity of the IUPAC codes^3^. For each *α*-cluster, we count first the number of occurrences of symbols of each read that we can associate with the occurrences of the same symbol of each genome appearing in the *α*-cluster and then we count the ambiguity symbols of each read (resp. genome) if they match a genome (resp. read) symbol that is associated with its code. Then, we sum these two values.

#### **Definition 2**

Let $\mathcal {C}_{\alpha }$ be the set of all the *α*-clusters associated with $\textsf {ebwt}(\mathcal {S})$. We define the *α*^eBWT^*-similarity* between two sequences $S_{i}\in \mathcal {R}$ and $S_{j}\in \mathcal {G}$ as the quantity

$$ \mathfrak{S}^{\text{eBWT}}_{\alpha}(S_{i},S_{j})=\sum_{x\in\mathcal{C}_{\alpha}} \left(\sum_{a\in\Sigma} \min\,\left(\text{occ}_{a}{(i,x)},\text{occ}_{a}{(j,x)}\right)+ \delta_{IUPAC}\right) $$

where occ_*a*_(*i*,*x*) (resp. occ_*a*_(*j*,*x*)) is the number of *a*-symbols belonging to *S*_*i*_ (resp. *S*_*j*_) in the *α*-cluster *x*, and *δ*_*IUPAC*_ is obtained as the sum of the number of matchings between the remaining IUPAC ambiguity symbols of *S*_*i*_ (resp. *S*_*j*_) with any remaining symbol associated with its code of *S*_*j*_ (resp. *S*_*i*_).

In the second approach, whose related measure we call *α*^DA^-similarity, we only consider the number of symbols from $S_{i}\in \mathcal {R}$ and $S_{j}\in \mathcal {G}$ appearing in the *α*-clusters, that is, we disregard the correspondence between the symbols *a*∈*Σ* from *S*_*i*_ and *S*_*j*_ in the *α*-cluster. In order to do this, we scan sequentially the corresponding positions of the *α*-clusters in $\textsf {da}(\mathcal {S})$ counting the colors of *S*_*i*_ and *S*_*j*_.

#### **Definition 3**

Let $\mathcal {C}_{\alpha }$ be the set of all the *α*-clusters associated with $\textsf {ebwt}(\mathcal {S})$. We define the *α*^DA^*-similarity* between two sequences $S_{i}\in \mathcal {R}$ and $S_{j}\in \mathcal {G}$ as the quantity

$$ \mathfrak{S}^{\text{DA}}_{\alpha}(S_{i},S_{j})=\sum_{x\in\mathcal{C}_{\alpha}} \min\,\left(\text{occ}{(i,x)},\text{occ}{(j,x)}\right) $$

where occ(*i*,*x*) (resp. occ(j,x)) is the number of symbols belonging to *S*_*i*_ (resp. *S*_*j*_) in the *α*-cluster *x*.

Note that $0 \leq \mathfrak {S}^{\text {eBWT}}_{\alpha }(S_{i},S_{j}) \leq \mathfrak {S}^{\text {DA}}_{\alpha }(S_{i},S_{j}) \leq \min (n_{i},n_{j})+1-\alpha $, where *n*_*i*_ (resp. *n*_*j*_) is the length of *S*_*i*_ (resp. *S*_*j*_). So, we can normalize the similarity values within the range [0,1] dividing them by min(*n*_*i*_,*n*_*j*_)+1−*α*.

Let $\mathcal {S}=\{S_{1}, \ldots, S_{r}, S_{r+1}, \ldots, S_{r+g} \}$. For each *i*=1,…,*r* and *k*=1,…,*g*, we build the matrix of similarity $\mathcal {M}_{\alpha }$ of dimension *r*×*g*, where

$$\mathcal{M}_{\alpha}[i][k]=\frac{\mathfrak{S}^{\text{eBWT}}_{\alpha}(S_{i},S_{j})}{\min(n_{i},n_{j})+1-\alpha}$$

for the first approach, and

$$\mathcal{M}_{\alpha}[i][k]=\frac{\mathfrak{S}^{\text{DA}}_{\alpha}(S_{i},S_{j})}{\min(n_{i},n_{j})+1-\alpha}$$

for the second approach.

#### **Example 3**

(running example) For *α*=2, both the *α*^eBWT^-similarity and the *α*^DA^-similarity between *S*_1_ and *S*_2_ are given by the sum 0+1+1+1+1+1=5, which by normalizing holds $\mathfrak {S}^{\text {eBWT}}_{2}(S_{1},S_{2})/8=\mathfrak {S}^{\text {DA}}_{2}(S_{1},S_{2})/8=0.625$.

On the other hand, the *α*^eBWT^-similarity between *S*_1_ and *S*_3_ is given by $\mathfrak {S}^{\text {eBWT}}_{2}(S_{1},S_{3})= 1+0+0+0+0+1=2$, by normalizing $\mathfrak {S}^{\text {eBWT}}_{2}(S_{1},S_{3})/8=0.250$. Note that, since the ambiguity symbol *R* is associated with *A* and *G*, we have one match in the last cluster (i.e., *R* matches with *G*). While the *α*^DA^-similarity is given by $\mathfrak {S}^{\text {DA}}_{2}(S_{1},S_{3})= 1+1+0+0+0+1=3$, which normalized is 0.375.

We observe that $\mathfrak {S}^{\text {DA}}_{\alpha }(S_{i},S_{j})$ differs from $\mathfrak {S}^{\text {eBWT}}_{\alpha }(S_{i},S_{j})$ and from the measure defined in [45], since it does not take into account whether a symbol is an ambiguous character or not, so we expect the classification w.r.t. $\mathfrak {S}^{\text {eBWT}}_{\alpha }(S_{i},S_{j})$ to be more precise and less sensitive than that w.r.t. $\mathfrak {S}^{\text {DA}}_{\alpha }(S_{i},S_{j})$, while computing $\mathfrak {S}^{\text {DA}}_{\alpha }(S_{i},S_{j})$ should be faster than $\mathfrak {S}^{\text {eBWT}}_{\alpha }(S_{i},S_{j})$. The experiments reported on both simulated and real datasets confirm these facts.

Both approaches of Step 2 allow to analyze *α*-clusters independently: indeed, we can analyze the clusters in $\mathcal {C}_{\alpha }$ one by one through $|\mathcal {C}_{\alpha }|$ iterations, or in parallel exploiting the fact that *α*-clusters are pairs of indices not overlapping.

**Single-read and Paired-end read collections —** LiME is designed to work either with single-read collection or with paired-end reads of any insert size. In addition, both the original sequences and their reverse complements must be considered in order to keep reads properly oriented (original reads’ strand directions are unknown). Paired-end reads (and their reverse complement) are treated initially as two single reads, so that they can overlap and align in several ways. Then, our tool exploits the pairing information during the classification step, which we describe in the next paragraph. Therefore, in case of single-read collections, we keep two read sets, $\mathcal {R}$ and $\mathcal {R}^{RC}$, with forward and reverse complement. After performing the procedures at Step 1 and Step 2 for the data structures of both $\mathcal {R} \cup \mathcal {G}$ and $\mathcal {R}^{RC}\cup \mathcal {G}$, to perform classification we use the information coming either from $\mathcal {R}\cup \mathcal {G}$ or from the reverse complement $\mathcal {R}^{RC}\cup \mathcal {G}$. On the other hand, if we have a paired-end read collection made of sets $\mathcal {R}_{1}$ and $\mathcal {R}_{2}$, the available information to perform the read classification comes from both paired-end reads and their reverse complements: thus, after performing the first two steps for the four input data structures ($\mathcal {R}_{1} \cup \mathcal {G}$, $\mathcal {R}_{1}^{RC}\cup \mathcal {G}$, $\mathcal {R}_{2}\cup \mathcal {G}$ and $\mathcal {R}_{2}^{RC}\cup \mathcal {G}$), we obtain four similarity matrices, denoted by $\mathcal {M}^{1F}_{\alpha }$, $\mathcal {M}^{1RC}_{\alpha }$, $\mathcal {M}^{2F}_{\alpha }$, and $\mathcal {M}^{2RC}_{\alpha }$, and to classify the pair of reads, we recollect the pairing information. Actually, our theoretical approach holds also if one uses a unique data structure for both mates and their reverse complements (without replicating the genomes) and if one uses the reverse complement of the genome set (rather than the read set).

**Step 3: classification—** The last step consists in assigning a unique provenance to any read in the input collection. In this step, one can specify a minimum taxonomic level (e.g., genome, species, genus or higher) that will be the minimum rank considered in the classification by LiME. Indeed, LiME can classify at higher levels by taking advantage of the taxonomic lineage of taxa.

To perform the assignment we need to take into account both strands (forward and reverse-complement) or, if a paired-end reads collection is available, we take both strands of each paired-end read.

Here, we consider the case where the input collection is paired-end (we have $|\mathcal {R}_{1}|=|\mathcal {R}_{2}|=r)$. Thus, for any index *i*, with 1≤*i*≤*r*, the entry $\overline {R}_{i}$ of a paired-end collection is associated with four different reads each one belonging to one of the sets $\mathcal {R}_{1}$, $\mathcal {R}_{1}^{RC}$, $\mathcal {R}_{2}$, $\mathcal {R}_{2}^{RC}$. Recall that, as output of Step 2, we obtained respectively the four similarity matrices $\mathcal {M}^{1F}_{\alpha }$, $\mathcal {M}^{1RC}_{\alpha }$, $\mathcal {M}^{2F}_{\alpha }$, and $\mathcal {M}^{2RC}_{\alpha }$, each one of size *r*×*g*, where *g* is the size of the genome set $\mathcal {G}$.

We remark that if we have a single-read collection, we can proceed similarly skipping the information about the mates $\mathcal {R}_{2}$ and $\mathcal {R}_{2}^{RC}$.

To assign each entry $\overline {R}_{i}$ to a taxonomic rank, we have to analyze the *i*-th row of each similarity matrix, denoted by $\mathcal {M}^{1F}_{\alpha }[\!i]$, $\mathcal {M}^{1RC}_{\alpha }[\!i]$, $\mathcal {M}^{2F}_{\alpha }[\!i]$ and $\mathcal {M}^{2RC}_{\alpha }[\!i]$.

Our classification strategy proceeds according to the difficulty in classifying any entry $\overline {R}_{i}$ by using first separately and then jointly the information belonging to an individual mate.

We set a threshold value *β* (0≤*β*<1) and we attempt to classify $\overline {R}_{i}$ only if there is at least one similarity value in $\mathcal {M}^{1F}_{\alpha }[\!i]$, $\mathcal {M}^{1RC}_{\alpha }[\!i]$, $\mathcal {M}^{2F}_{\alpha }[\!i]$, $\mathcal {M}^{2RC}_{\alpha }[\!i]$ greater than *β*. Such a parameter *β* may affect the classification accuracy: it is easy to see that decreasing the value of *β* implies a major number of reads that may be classified (increasing sensitivity) at the cost of a larger probability of error (decreasing precision).

For each $\overline {R}_{i}$, in a first phase, we compute the maximum value $M_{i}^{x}$, with *x*=1*F*, 1*RC*, 2*F*, 2*RC*, in each row $\mathcal {M}^{x}_{\alpha }[\!i]$ and we take the maximum *M*_*i*_ of these values, i.e., $M_{i}=\max M_{i}^{x}$. In general, we only consider the genomes in $\mathcal {G}=\{G_{1},\ldots, G_{g}\}$ that approximately reach, with the entry $\overline {R}_{i}$, a similarity value equal to *M*_*i*_ (i.e., with tolerance of 0.02). If *M*_*i*_≤*β* the entry $\overline {R}_{i}$ is said to be *not classified*.

If the specified minimum taxonomic rank is genome, the entry $\overline {R}_{i}$ is said to be *classified* to *G*_*j*_ only if there exists a unique *j* such that $\mathcal {M}^{x}_{\alpha }[\!i][\!j]$ is approximately equal to *M*_*i*_, whereas it is classified to a taxon *T* if all genomes *G*_*j*_, such that $\mathcal {M}^{x}_{\alpha }[\!i][\!j]$ is approximately equal to *M*_*i*_, belong to the same taxonomic unit *T* within the specified minimum taxonomic rank. In the case there exist more genomes *G*_*j*_ (1≤*j*≤*g*), with $\mathcal {M}^{x}_{\alpha }[\!i][\!j]$ approximately equal to *M*_*i*_ belonging to distinct taxonomic units, $\overline {R}_{i}$ is said to be *ambiguous*. In the next paragraph, we join the paired-end information for a re-examination of ambiguous reads.

**Re-examination of ambiguous reads–** In this second phase, we only consider ambiguous entries. For each entry $\overline {R}_{i}$, we build the set $\mathcal {I}_{i}$ of indices *q* such that, for at least one *x*=1*F*, 1*RC*, 2*F*, 2*RC*, $\mathcal {M}^{x}_{\alpha }[\!i][\!q]$ is approximately equal to the maximum *M*_*i*_ (i.e. with tolerance of 0.02).

For each genome *G*_*j*_ such that $j \in \mathcal {I}_{i}$, we take the maximum $M_{i}^{'}$ obtained by summing both $\mathcal {M}^{1F}_{\alpha }[\!i][\!j]$ and $\mathcal {M}^{1RC}_{\alpha }[\!i][\!j]$ with their corresponding mate. If we have only one genome *G*_*k*_ (with $k \in \mathcal {I}_{i}$) corresponding to the maximum value $M_{i}^{'}$, we are able to assign $\overline {R}_{i}$ to *G*_*k*_ or simply to its corresponding taxon at the specified rank. Whereas $\overline {R}_{i}$ is classified to a taxon *T* if all such genomes *G*_*k*_ (with $k \in \mathcal {I}_{i}$) that reach $M_{i}^{'}$ belong to the same taxonomic unit *T* within the specified minimum taxonomic rank. Otherwise, $\overline {R}_{i}$ still remains *ambiguous*, and we proceed with the last classification phase, as follows.

Then, we compute the maximum $M_{i}^{''}$ obtained by summing both $\mathcal {M}^{1F}_{\alpha }[\!i][\!j]$ and $\mathcal {M}^{1RC}_{\alpha }[\!i][\!j]$ with their corresponding mate, for all *j*=1,…,*g* (hence not only for $j \in \mathcal {I}_{i}$). We select the indices *h* (1≤*h*≤*g*) such that the sum between either $\mathcal {M}^{1F}_{\alpha }[\!i][\!j]$ and its mate, or $\mathcal {M}^{1RC}_{\alpha }[\!i][\!j]$ and its mate, is $M_{i}^{''}$. The procedure then follows as above.

However, if the entry $\overline {R}_{i}$ still remains ambiguous, then we are not able to classify. Thus, eventually we can either leave $\overline {R}_{i}$ as ambiguous, if the taxonomic level of classification is fixed, or classify it at higher ranks by taking advantage of the taxonomic lineage of taxa.

Note that in both re-examinations, we take the strand that gives the maximum similarity score, rather than using the summed score of both strands, as it might create a bias in the read classification.

## Results

In this section, we describe and test our prototype tool, named LiME [49], implemented in C++. It is arranged as a pipeline that runs the three steps described in the previous section. We distinguish two approaches according to the used similarities: LiME^eBWT^ when $\mathfrak {S}^{\text {eBWT}}_{\alpha }$ is used, and LiME^DA^ when $\mathfrak {S}^{\text {DA}}_{\alpha }$ is used.

In order to evaluate the performance of LiME, we have designed a series of tests with various paired-end read datasets: on both simulated and real datasets. Moreover, to guarantee a fair evaluation among tools, in these experiments, we used customized uniform reference databases with the aim to eliminate any confounding effects of differences between default databases (as specified in [50]).

We compare LiME with five sswell-known tools recently introduced that allow to build customized databases: CLARK-S [5], that uses spaced *k*-mers rather than simple *k*-mers; Centrifuge [4], that adapts the data structures of read-mapping algorithms based on the BWT and the FM-index [16] (such as Bowtie [51], which provides very fast alignment with a relatively small memory footprint); Kraken 2 [7], that is based on a probabilistic, compact hash table to map minimizers to lowest common ancestors taxa; TaxMaps [6], that is a taxonomic mapping tool, where the reads are mapped in single-end mode to an indexed database using GEM mapper [17]; Magic-BLAST [18], that is a tool for mapping DNA or RNA sequences against a whole genome or transcriptome^4^. In order to align reads, Magic-BLAST uses a BLAST database that can be customized based on a desired set of sequences. However, unlike Nucleotide-nucleotide BLAST (BLASTN), Magic-BLAST is aware of paired-end sequencing and the best alignments are selected based on the alignment quality of the pair.

### On simulated datasets

**Dataset description.** For the experiments on simulated data, we have used the collections made available by the benchmarking analysis carried out in [14]. Indeed, the authors of [14] evaluate the most widely used tools for metagenome classification by testing them on complex and realistic datasets, which have been designed ad-hoc for this benchmarking analysis and made publicly available [52].

In particular, we perform the validation of our approach by using two sets of metagenomes, randomly selected, among those provided by Lindgreen et al. [14]: the two datasets of paired-end reads *setA2* and *setB2* reproduce the size, complexity and characteristics of real metagenomic samples containing around 20 millions of sequences of length 100 belonging to 17 different phyla. Some phyla are included in equal proportions, whereas some others vary more substantially between the two sets (see ([14], Supplementary Table S1)).

Moreover, as to test the reliability of the tools, each dataset has been enhanced with a set of simulated negative control sequences to mimic sequences from “unknown” organisms (i.e., their genomes are not present in the reference database) that are likely to appear in metagenome samples – see [14] for further details. Each of these negative control subsets added to *setA2* and *setB2* includes around 5 million of random shuffled reads.

We precise that the original datasets, downloadable from [14], are not exactly the datasets *setA2* and *setB2* we use for our evaluations [53]. In fact, we first removed a group of reads associated with the phylum of Eukaryotes whose species provenance was not specified in [14]. Second, since we use a custom database and up-to-date taxonomy data (such as taxonomy id, or accession numbers) downloaded from the NCBI website [54], we preferred not to include in sets *setA2* and *setB2* groups of reads associated with 3 genomes whose entries in the NCBI database have been indicated as expiring.

The reference database $\mathcal {G}$ we use for these experiments comprises 930 genomes from 686 species belonging to 17 phyla as indicated in ([14], Supplementary Table S2).

**Validation step.**

In our experiments, we consider only read assignments at species level, and thus all non-random reads assigned to higher taxonomic levels (i.e., more species could be assigned to them) are counted in *FN*.

As the provenance of the simulated reads is known, we denote by *TP* (true positives) the number of reads correctly classified (i.e., assigned to their right species), by *FP* (false positives) the number of reads erroneously classified, and by *FN* (false negatives) the number of reads unassigned, from which we can calculate the quality metrics: sensitivity SEN, precision PREC, and the F1 score that is the harmonic mean between sensitivity and precision,

$$\text{SEN}=\frac{TP}{TP+FN},\quad\text{PREC}=\frac{TP}{TP+FP},\quad\text{F1}=\frac{2TP}{2TP+FP+FN}.$$

For simulated negative control sequences that do not belong to any known species, we can set *TN* as the number of random reads that are correctly not mapped to any species, and calculate the specificity

$$\text{SPEC}=\frac{TN}{t},\text{ where \textit{t} is the total number of random shuffled reads.}$$

**Experiments**

**Fig. 2:**
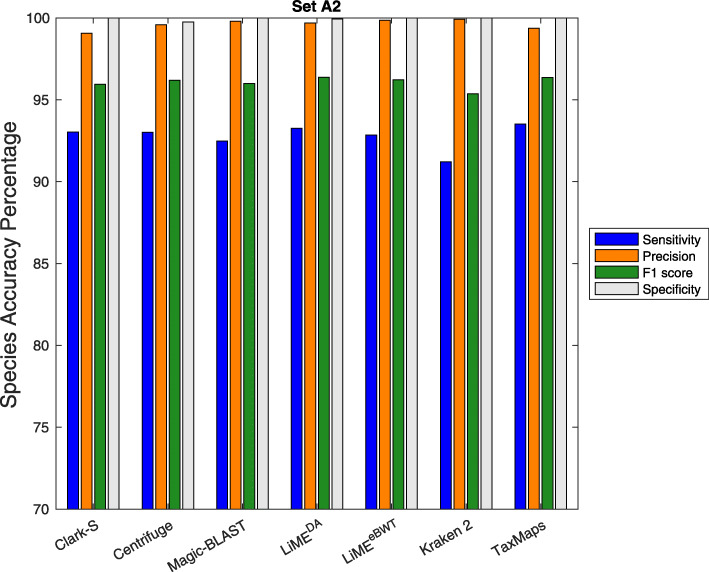
Comparison between LiME and other sequence classification tools. The results here are shown for Sensitivity, Precision, F1-score and Specificity as evaluated at the species rank on the dataset *setA*2. Full results are available in Additional file 1: Table S1

**Fig. 3:**
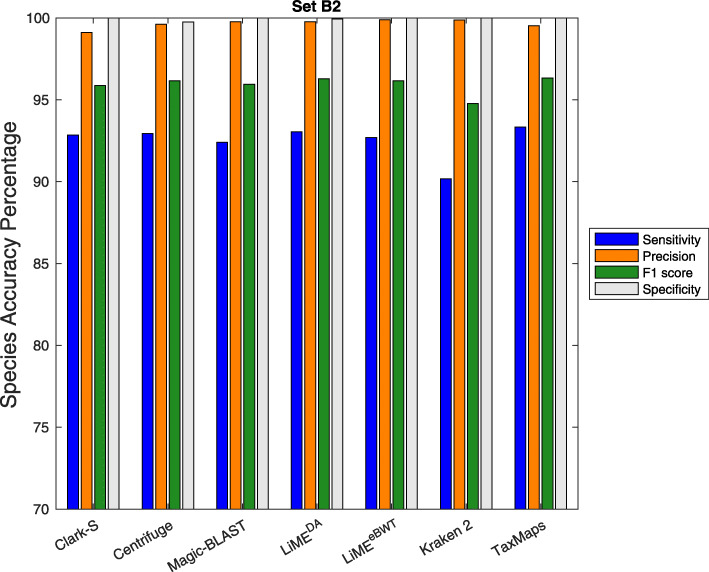
Comparison between LiME and other sequence classification tools. The results here are shown for Sensitivity, Precision, F1-score and Specificity as evaluated at the species rank on the dataset *setB*2

Our tool is able to classify the reads to several taxonomic levels such as genomes, species or phylum. For the experiments reported in Figs. 2 and 3, we set the taxonomic rank of classification to species.

To run Magic-BLAST, Centrifuge, Kraken 2 and TaxMaps we use the default values provided for paired-end reads. CLARK-S can run with default values only and the results are filtered by using the recommended option –highconfidence (e.g., assignments with confidence score <0.75 and gamma score <0.03 are discarded).

For LiME, we set the minimum length of the common context *α*=16, since the length of each paired-end read is 100, and we provide results for minimum similarity scores *β*=0.25.

**Fig. 4:**
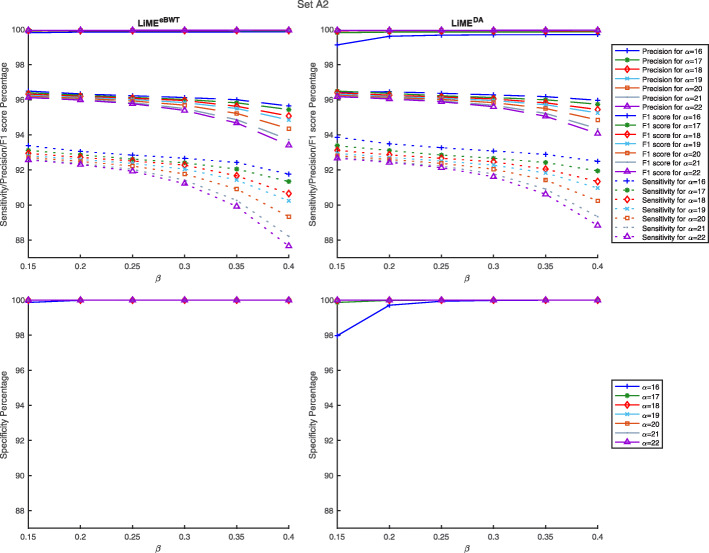
Comparison of accuracy by using on dataset *setA*2. The results here are shown for sensitivity, precision, F1-score and Specificity as evaluated at the species using various parameter values on the dataset *setA*2. Full results are available in Additional file 1: Table S1

**Fig. 5:**
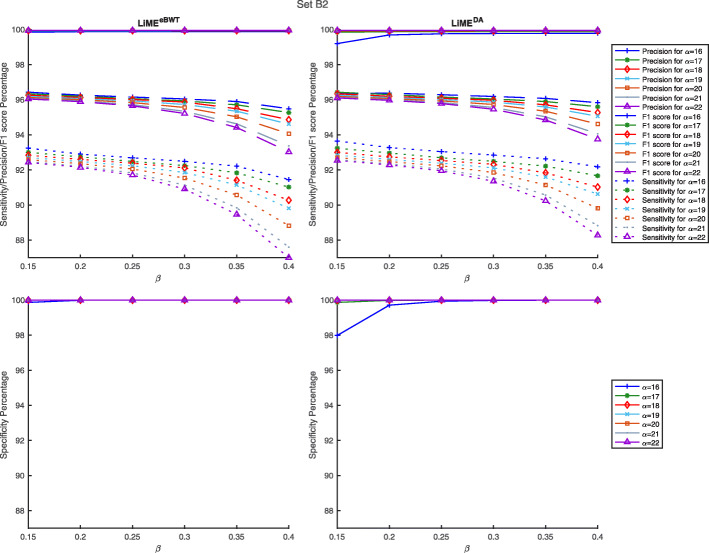
Comparison of accuracy by using on dataset *setB*2. The results here are shown for sensitivity, precision, F1-score and Specificity as evaluated at the species using various parameter values on the dataset *setB*2. Full results are available in Additional file 1: Table S1

We additionally processed these datasets with different parameters (see “Discussion” section, and Figs. 4 and 5) and show that for fixed *α*, the greater the value *β* is, the more the sensitivity decreases and the precision increases.

In Figs. 2 and 3, we report the classification results for the simulated datasets *setA*2 and *setB*2. It is possible to observe that both LiME^eBWT^ and LiME^DA^, together with Magic-BLAST and Kraken 2, show higher precision than taxMaps, Clark-S and Centrifuge on both datasets. Moreover, both LiME^eBWT^ and LiME^DA^, together with Clark-S, Centrifuge, and taxMaps, achieve higher sensitivity than Kraken2 and Magic-BLAST on both datasets. The specificity and the F1 score achieved by both LiME^eBWT^ and LiME^DA^ are comparable or superior to those of the other classifiers.

Finally, there is experimental evidence that taking into account the eBWT symbols (LiME^eBWT^) rather than considering only the provenience of the symbols (LiME^DA^) leads to achieve a higher precision at the cost of a lower sensitivity.

### On real datasets

**Dataset description.** For the experiments on real data, we have used a paired-end read collection from the Human Microbiome Project (HMP). The real metagenome *SRR1804065* is a DNA tool sample of a female participant generated by using Illumina sequencing, that has been previously studied in [9, 55]. The paired-end reads have length 100 bps and their total number in the original dataset was 21,873,781. Since the “ground truth” is not available for a real metagenome and a large number of reads may belong to unknown species, we first filtered this dataset by using BLAST: we mapped reads against the whole nucleotide sequence database with a sequence identity of 98%, and discarded the pairs of reads that do not map to any genome. The resulting dataset *SRR1804065* comprises 5,654,624 paired-end reads.

The reference database $\mathcal {G}$ we use for this experiment comprises 3,423 genomes from 1,499 species belonging to 42 phyla.

**Validation step.**

Because a real metagenomic dataset is from real sequencing experiments, we cannot be certain of the true taxonomic origin of each individual fragment, and consequently we cannot report sensitivity and precision for this dataset. However, we evaluate the concordance between the reads’ classifications performed by the tested tools at the species level. First, we report for each classifier the following quantities: (a) the number of reads that are assigned at the species level, (b) the number of reads with a classification at higher taxonomic levels than species, (c) the number of reads that are not classified.

Furthermore, with the purpose of comparing our method to the state-of-the-art tools, we represent the reads classified at the species level as set elements. Then, to measure the similarity of classification between two tools, we compute the similarity between the two sets of classified reads by using the *Jaccard similarity* coefficient.

The Jaccard coefficient *J*(*A*,*B*) is defined as the size of the intersection divided by the size of the union of the two sets *A* and *B*, i.e., |*A*∩*B*|/|*A*∪*B*|.

For our purposes, the set *A* (resp. *B*) is the set of reads classified by a tool (resp. by a competitor) at the species level, thus we denote by *t* the total number of reads classified joining the two classifications. On the one hand, we can consider the identifiers of the reads, so that the intersection *I*_*id*_=*A*∩*B* is given by those reads that are classified by both tools at the species level. On the other hand, we can consider the species assigned to those reads, so that the intersection *I*_*as*_=*A*∩*B* is given by those reads that are assigned by both tools to the same taxonomic unit. In order to quantify the concordance between the two tools on the basis of the Jaccard coefficient, we calculate the two agreement rates

$$r_{id}=\frac{|I_{id}|}{t} \;\;\text{ and }\;\;r_{as}=\frac{|I_{as}|}{t}.$$

**Experiments** Also for the real metagenome, in order to guarantee a fair evaluation, we use a customed reference database for all the tested tools.

As for simulated reads, the taxonomic level we choose to classify reads is the deep level of species. In fact, although our tool can classify reads at different taxonomic levels, we fixed the taxonomic level to fairly compare our tools to the others.

For Magic-BLAST we use default values provided for paired-end reads, and for CLARK-S we use the recommended option –highconfidence to filter the classification results. Centrifuge, Kraken 2 and taxMaps use default values, while LiME uses the parameters *α*=16 and *β*=0.25, as we did for simulated reads.

Table 1 reports for each tool the number of reads that are assigned to a particular species, the number of reads that are assigned to higher taxonomic levels, and the number of unclassified reads. Note that CLARK-S classifies reads only at a given taxonomic level, thus the number of reads assigned to higher ranks is 0.

**Table 1:** Analysis results of the real metagenome (*SRR1804065*) at species level

*SRR1804065*	Reads	%	Reads	%	Reads	%
	assigned at		assigned at		unassigned	
	species level		higher rank			
LiME^eBWT^	4,524,097	80.007	1,019,702	18.033	110,825	1.960
*α*=16, *β*=0.25						
LiME^DA^	4,563,319	**80.701**	991,361	17.532	99,944	1.767
*α*=16, *β*=0.25						
Kraken 2	4,497,709	79.540	1,085,413	19.195	71,502	1.264
taxMaps	4,331,521	76.601	1,240,035	21.930	83,068	1.469
CLARK-S	4,414,957	78.077	0	0	1,239,667	21.923
Centrifuge	4,461,655	78.903	1,170,321	20.697	22,648	**0.401**
Magic-BLAST	4,392,446	77.679	1,081,523	19.126	180,655	3.195

Best scores are in bold

On the real metagenome, our approach achieves the highest number of classified reads at the species level: as shown in Table 1, both LiME^eBWT^ and LiME^DA^ classify a number of reads larger than CLARK-S, Centrifuge, Kraken 2, Magic-BLAST and taxMaps, while the smallest number of unclassified reads is reached by Centrifuge.

In Table 2, we show numerically how much the classification results differ among pairs of tools. In particular, we perform a direct comparison between our tool and the others tools, for completeness we also report the comparison between Magic-BLAST and the others tools, since we built the customized reference database by means of BLAST. However, we do not report a direct comparison between other pairs of tools, as it is out of the scope of this work. More precisely, in Table 2, we compare the classification results of both LiME^eBWT^ and LiME^DA^ to those of CLARK-S, Centrifuge, Magic-BLAST, Kraken 2 and taxMaps. By comparing LiME^eBWT^ and Magic-BLAST, it results not only that the percentage of individual reads classified by both tools is the 95.2*%* of all classified reads, which is the highest value in Table 2, but also that the 93.6*%* of all classified reads is assigned to the same taxon by LiME^eBWT^ and Magic-BLAST. All the other percentages reported in Table 2 are strictly lower.

**Table 2:** Classification comparison on real metagenome (*SRR1804065*) by using the agreement rates *r*_*id*_=|*I*_*id*_|/*t* and *r*_*as*_=|*I*_*as*_|/*t*

	*t*	|*I*_*id*_|	*r*_*id*_(%)	|*I*_*as*_|	*r*_*as*_(%)
LiME^eBWT^ and Kraken 2	4,631,891	4,389,915	94.776	4,270,991	92.208
LiME^eBWT^ and taxmaps	4,619,370	4,236,248	91.706	4,171,501	90.305
LiME^eBWT^ and CLARK-S	4,898,446	4,039,348	82.462	3,971,990	81.087
LiME^eBWT^ and Centrifuge	4,667,402	4,318,350	92.521	4,208,552	90.169
LiME^eBWT^ and Magic-BLAST	4,566,885	4,349,658	**95.243**	4,274,772	**93.604**
LiME^DA^ and Kraken 2	4,657,542	4,403,486	94.545	4,274,384	91.773
LiME^DA^ and taxmaps	4,648,287	4,246,553	91.357	4,175,008	89.818
LiME^DA^ and CLARK-S	4,924,196	4,054,080	82.330	3,976,705	80.758
LiME^DA^ and Centrifuge	4,694,705	4,330,269	92.237	4,212,149	89.721
LiME^DA^ and Magic-BLAST	4,602,062	4,353,703	94.603	4,276,833	92.933
Magic-BLAST and Kraken 2	4,614,673	4,275,482	92.650	4,186,614	90.724
Magic-BLAST and taxmaps	4,512,340	4,211,627	93.336	4,188,458	92.822
Magic-BLAST and CLARK-S	4,823,733	3,983,670	82.585	3,925,157	81.372
Magic-BLAST and Centrifuge	4,647,144	4,206,957	90.528	4,086,148	87.928

Best scores are in bold

**Fig. 6:**
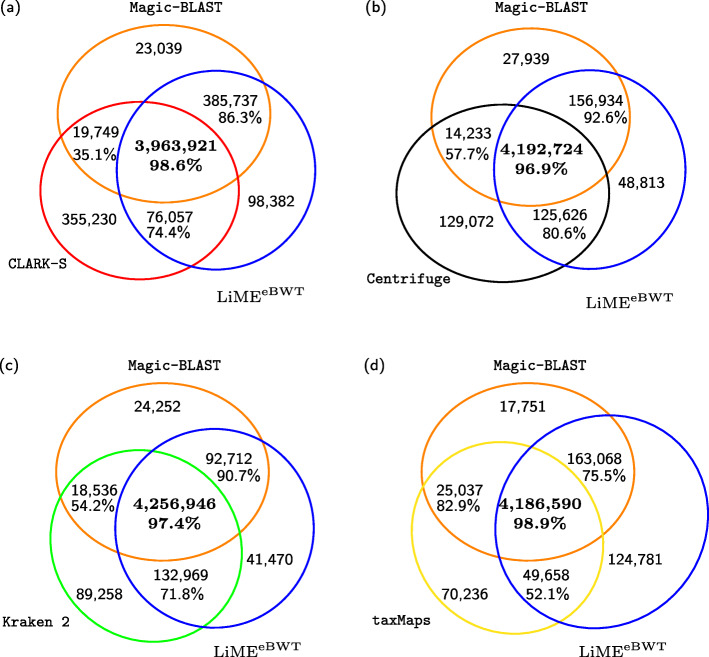
Graphical representations of the results in Table 2: (**a**) related to LiME^eBWT^, Magic-BLAST and CLARK-S, (**b**) related to LiME^eBWT^, Magic-BLAST and Centrifuge, (**c**) related to LiME^eBWT^, Magic-BLAST and Kraken 2, (**d**) related to LiME^eBWT^, Magic-BLAST and taxMaps. The black numbers inside each circle if summed give the number of reads classified by the corresponding tool. The percentage under each number denotes the percentage of classified identically reads among two/three tools (see Additional file 1: Table S5)

Analogously to the experiments on simulated data, our strategy using the first approach (LiME^eBWT^) classifies a smaller number of reads than the second approach (LiME^DA^), however the agreement rates *r*_*id*_ and *r*_*as*_ in Table 2 between Magic-BLAST and our strategy are higher using the first approach. In Fig. 6, thus, we report only a more in-depth analysis of the classification differences between LiME^eBWT^ and the other tools, however similar observations can be deduced for LiME^DA^ by looking at Table 2. In Fig. 6, we evaluate the classification concordance comparing LiME^eBWT^, Magic-BLAST and another tool. In each picture, the sets of classified reads are represented by a Venn diagram: the reads lying in the intersection of two/three sets are those classified by both/all tools. The number of individual reads classified by all the three tools is greater in Fig. 6c when comparing LiME^eBWT^, Magic-BLAST and Kraken 2, while comparing LiME^eBWT^, Magic-BLAST and CLARK-S such number is the smallest (see Fig. 6a).

Moreover, we notice that the highest number of reads that are classified by one single tool only is registered for CLARK-S (see Fig. 6a).

In Fig. 6, we report, in addition, the percentage of reads with a concordant classification among both/three tools: the assignments performed by LiME^eBWT^ are closer to Magic-BLAST (as shown in Table 2), without however largely differing from those performed by all the other tools. In fact, according to Fig. 6a, d among the reads that are classified by all the three tools, around 98% of them are assigned to the same taxon by all. Overall, for the real metagenome the agreement rates among the tested tools are high, and LiME classification meets that of state-of-the-art classifiers, such as CLARK-S, Centrifuge, Kraken 2 and taxMaps.

## Discussion

In this section we analyze the impact of the parameters *α* and *β* and discuss the data structures and the resource usage.

**Parameter sweeps** In Figs. 4 and 5, we looked at parameters relating to the minimum length *α* of the context in Step 1 and to the threshold value *β* in Step 3. The parameter sweep analyzed values in the interval [16,22] for *α* and in the interval [0.15,0.40] for *β*.

We note that, in both approaches, the parameter sweeps give approximately the same, near-optimal levels of accuracy (see Additional file 1: Table S2, for further details). The experimental results confirm that, fixed *α*, the parameter *β* affects the classification accuracy: indeed, decreasing the value of *β* implies a major sensitivity at the cost of a minor precision. So, the trend of the sensitivity curve decreases, while that of precision increases reaching almost 100%. This suggests performance is not overly sensitive to particular parameter settings. From the analysis of the parameter sweep results and from reported experimental results during the comparisons with other tools, it seems that *α*=16 and *β*=0.25 is a good sensitivity-precision trade-off.

**Observations on the data structures.**

We recall that our tool takes as input the files containing $\textsf {ebwt}(\mathcal {S})$, $\textsf {da}(\mathcal {S})$ and $\textsf {lcp}(\mathcal {S})$ of the collection $\mathcal {S}$ of reads and reference genomes. This task can be achieved using, for example one of the following tools, BCR [32, 56], eGSA [47, 57], gSACA-K [58, 59], GAP [30] or eGAP [34, 60]. It is interesting to note that the data structures for the genome database used by our strategy can be built once and stored, and then, for each new experiment, we can build the data structures for the read collection and merge the two data structures. More precisely, as the set $\mathcal {G}$ of genomes is the same for each experiment, we can build the data structures of $\mathcal {G}$ only once, by using eGSA for instance, and keep them stored. Then, for each new experiment, we can build the data structures for each read collection $\mathcal {R}$, for instance, by using BCR (a tool for very large collection of short reads), and finally, merge the two data structures, by using eGAP, and obtain those for the entire collection $\mathcal {S}$. On the other hand, by exploiting the mathematical properties of the permutation associated with the eBWT and LCP array, by using BCR [31, 32], we could build once the data structures for $\mathcal {G}$ and then update with the symbols from $\mathcal {R}$ (hence, without constructing the eBWT of reads) in order to obtain the data structures for $\mathcal {S}$. However, to find the best method for building our data structures is not in the aim of this paper.

We also remark that the data structures used by our strategy are intrinsically dynamic: by taking advantage of the inherent properties of the eBWT and LCP array (see, in particular, ([31], Remark 3.6)), the collection $\mathcal {S}$ can be modified by inserting or removing single sequences. This may allow to modify the read dataset by inserting or removing a group of reads, or to update the reference database with newly arranged set of genomes, allowing us to modify *α*-clusters accordingly.

Moreover, we note that in the recent literature there are several papers with the aim of introducing new lightweight and parallel computational strategies for building the data structures we use in our tool, see for instance [33, 35].

In this paper, we use algorithm eGAP [34, 60] to merge $\textsf {ebwt}(\mathcal {R})$ and $\textsf {da}(\mathcal {R})$ (built by BCR tool) with $\textsf {ebwt}(\mathcal {G})$ and $\textsf {da}(\mathcal {G})$ (built by eGSA tool) and obtain $\textsf {ebwt}(\mathcal {S})$ and $\textsf {da}(\mathcal {S})$, so that the array $\textsf {lcp}(\mathcal {S})$ is given as a by-product of the algorithm. In particular, by using the option –trlcp (see [60]), one can compute $\textsf {lcp}(\mathcal {S})$ with values truncated in *k*, where *k* is the longest string length in the read collection. This merging procedure performs *O*(*N*×*k*) sequential scans. In order to use eGAP for our scope, we can set any *k*>*α* to minimize the number of steps.

**Resources** The construction of the required data structures is independent of our method, so one can use any strategy according to the resources available, preferring for example a tool that works in internal memory rather than in external memory, or vice-versa. In addition, such data structures also allow to efficiently compress the original files (FASTA or FASTQ), see for instance [61, 62]. Note that, the other tools (Centrifuge, CLARK-S, Kraken 2, taxMaps and Magic-BLAST) build ad-hoc data structures for the database used in the classification. For instance, CLARK-S needs about 120 GB of RAM for the database used for its classification of the simulated datasets, while Centrifuge, Kraken 2 and taxMaps need about 10 GB of RAM. Moreover, some tools require some information to be specified at the time of building a database, for instance Clark-S requires to set the rank level and Kraken 2 requires to set the *k*-mer length. Thus, they do not have the same flexibility in choosing how to build these data structures and the same independence from the parameter sweeps as LiME.

Classification experiments were performed by using a DELL PowerEdge R630 machine, 24-core machine with Intel(R) Xeon(R) CPU E5-2620 v3 at 2.40 GHz, with 128 GB of shared memory. The system is Ubuntu 14.04.2 LTS.

Regarding the time of classification steps, Kraken 2 is the fastest tool, while Magic-BLAST and taxMaps are the slowest ones (taxMaps took about 13 hours for classifying the simulated dataset *setA*2). Full results for the dataset *setA*2 are available in Additional file 1: Table S3.

Observe that for the classification of the dataset *setA*2, Clark-S is the tool that requires the most internal memory. While the internal memory used by LiME is about 19,034 MB for the similarity matrix and 786 MB for the other auxiliary data structures.

Moreover, we observe that LiME, unlike other methods, processes all the reads at the same time, so that in the current implementation we need to keep in internal memory the whole similarity matrix. If one wanted to run our tool on a system without enough RAM to store the similarity matrix, one could either store that matrix on an external file or one could build the list of the clusters for all the reads, and then consider one read per time analyzing only the clusters that contain symbols of that read. In the latter case, the required time for Step 2 and Step 3 of LiME^eBWT^ is a few milliseconds and the maximum resident set size is only 16,240 KB. Full results for the dataset *setA*2 are available in Additional file 1: Table S3.

So, on the basis of the available resources, one could choose the number of reads to be compared simultaneously, in order to get the desired time-memory trade-off.

Finally, since our method scans sequentially the required data structures, we could analyze unknown sequences of very large collections by using mainly external memory and reduce the internal memory usage.

The multi-threading version of our tool exploits the fact that our strategy allows a certain degree of parallelization thanks to the analysis of clusters that is independent of each other, so each thread can handle distinct parts of the input files by reading it through local buffers (Additional file 1: Table S4).

Moreover, we noticed that LiME^DA^ is faster than LiME^eBWT^. This experimental evidence can be easily understood, as LiME^DA^ saves time scanning only the DA, rather than both DA and eBWT during the cluster analysis. More specifically, with respect to our three-step method, LiME^DA^ is time-saving concerning the completion of the second step (as the first and the third step are the same for both approaches). However, the execution time of the third step largely depends on the number of not-null similarity scores calculated during the second step. Thus, the higher the sensitivity is, the more likely the execution time of the third step could increase.

## Conclusion

In this paper, we present a versatile, alignment-free and lightweight tool for metagenomic classification, named LiME, that, by sequentially scanning fundamental string data structures (eBWT, LCP and DA) allows us to efficiently identify the genome to which each read belongs.

Our method is based on two possible approaches: the first approach LiME^eBWT^ takes into account the different symbols in eBWT that precede a common context between the read and the genome that we are comparing. The second approach LiME^DA^ takes into account only the colors in DA of each read and the genome that we are comparing. Experiments on both simulated and real datasets corroborate our intuition that the first approach is more precise and less sensitive than the second approach, while keeping high precision and sensitivity in both cases.

Moreover, we compare LiME (both approaches) with the state-of-the-art tools: Magic-BLAST, CLARK-S, Centrifuge, Kraken 2 and TaxMaps, which have recently been introduced.

In the experiments, we focused the attention on species level classification, but LiME can also work at higher taxonomic levels such as genus, family, class or phylum. Further experiments at phylum level on simulated datasets show that the relative phylum abundance estimated by LiME meets the dataset composition designed in [14] with very high precision. More precisely, LiME^eBWT^ on *setA*2 (resp. *setB2*) has 95.55*%* (resp. 95.95*%*) of sensitivity, and thus the F1 score achieved is 97.66*%* (resp. 97.91*%*). In particular, we obtain only 129,470 (resp. 40,895) ambiguous reads and 824,036 (resp. 779,515) not classified reads and we correctly classify 20,478,036 (resp. 19,419,539) in *setA2* (resp. *setB2*).

For the real metagenome dataset, as the “ground truth” is not available and a large number of reads may belong to unknown species, we first filtered the downloaded dataset by using BLAST and extracted information to build a customized reference database. Since Magic-BLAST is based on BLAST, we may consider the results of Magic-BLAST as a benchmark for the classification results. We can thus observe that only 42,788 reads of the considered real dataset classified by Magic-BLAST are not classified by LiME^eBWT^. Our tool classifies, indeed, the same reads as the aligner Magic-BLAST for the 95.2*%*. Whereas CLARK-S, (resp. Centrifuge, Kraken 2, and TaxMaps) fails to classify 408,776 (resp. 185,489, and 116,964, and 180,819) reads, which on the contrary are classified by Magic-BLAST. Furthermore, our tool assigns 93.6*%* of the reads to the same taxons as the aligner Magic-BLAST does.

Finally, we observe that the notion of LCP-interval [63] is a particular *α*-cluster (*pS*,*pE*) in which at least an index *i*, *pS*<*i*≤*pE*, is equal to *α*. Moreover, there exist several methods that are based on clustering of eBWT symbols with [62, 64, 65] or without the LCP array [21, 23]. Unlike these methods based on the partitioning of the LCP values, we do not impose any constraint on the *α*-cluster size.

In conclusion, we believe that our tool can be useful in a variety of applications both in metagenomics and in genomics.

## Supplementary information

**Additional file 1** **Table S1**. Classifier comparison of accuracy on simulated data. **Table S2**. Comparison of LiME accuracy using various parameter values **Table S3**. Comparison of computational performance. **Table S4**. Thread scaling evaluation results. **Table S5**. Microbiome comparison results

## Data Availability

The tool LiME is freely available for academic use at https://github.com/veronicaguerrini/LiME. Information to download the datasets used and analysed in the current study is available in the Datasets directory of the same Github repository. The datasets can also be available from the corresponding author on reasonable request.

## Abbreviations

NGS: Next-generation sequencing
BWT: Burrows-wheeler transform
eBWT: Extended burrows-wheeler transform
DA: Document array
LCP: Longest common prefix
LiME: Lightweight metagenomics via eBWT
BLAST: Basic local alignment search tool
IUPAC: International union of pure and applied chemistry
LCA: Lowest common ancestor
HMP: Human microbiome project
FP: False positives
TP: True positives
FN: False negatives
TN: True negatives

## Footnotes

1 More formally, a spaced seed *S* (or just a seed) is a binary string of length *k*, where the symbol ‘1’ requires a match in that position, while a symbol ‘0’ allows for “don’t care”. A spaced seed is characterized by its length *k* and by its weight *W*<*k*, which is the number of 1s in the string. A spaced seed always begins and ends with a 1.

2 The IUPAC code has defined a standard representation of DNA bases by single characters that specify either a single base (e.g., *G* for guanine, *A* for adenine) or a set of bases (e.g., *R* for either *G* or *A*).

3 We clarify that this measure differs from the measure introduced in [45], since in the preliminary work the ambiguity symbols could match with any symbol, as if they were placeholders.

4 A transcriptome is a collection of RNA molecules derived from genes, whose biological information is required by the cell at a particular time.
